# Barriers and facilitators of integrating cervical cancer screening into routine HIV care and acceptability of self-sampling for HPV DNA testing among people with HIV: an exploratory study in southern Ghana

**DOI:** 10.3389/fpubh.2025.1679325

**Published:** 2025-12-01

**Authors:** Evelyn Yayra Bonney, Emmanuel Frimpong Gyekye, Jonathan Klutse, Dzidzor Aku Attoh, Yeena Abla Tay, Nicholas Ekow Thomford, Mary Amoakoh-Coleman, Kwadwo Ansah Koram, George Boateng Kyei, Collins Stephen Ahorlu

**Affiliations:** 1 Department of Virology, Noguchi Memorial Institute for Medical Research, College of Health Sciences, University of Ghana, Accra, Ghana; 2 Department of Epidemiology, Noguchi Memorial Institute for Medical Research, College of Health Sciences, University of Ghana, Accra, Ghana; 3 University of Cape Coast School of Medical Sciences, Cape Coast, Ghana; 4 Medical and Scientific Research Centre, The University of Ghana Medical Center, Accra, Ghana; 5 Department of Medicine, Washington University School of Medicine in St. Louis, St. Louis, MO, United States

**Keywords:** access, cervical cancer, Ghana, HIV, provider-sampling, screening, self-sampling, WLWH

## Abstract

**Background:**

Cervical cancer poses a substantial global health challenge, disproportionately affecting low- and middle-income nations. Women living with HIV are disproportionately affected compared to the general population. Our study explored the acceptance and preferences of participants regarding cervical cancer screening procedures during their HIV clinic visits.

**Methods:**

Using the Consolidated Framework for Implementation Research (CFIR) to inform questions, we conducted semi-structured in-depth qualitative interviews with 85 people with HIV (PWH), across six southern Ghana sites, to identify factors that could promote or impede the integration of cervical cancer screening into HIV care in Ghana. We also explored the acceptability of self-sampling versus provider-sampling among respondents. Thematic analysis was performed on the data using the MAXQDA software.

**Results:**

Most participants demonstrated a strong willingness to participate in cervical cancer screening, were motivated by the desire to know their health status, protect themselves, benefit from early detection and access timely treatment. We identified potential for early detection and treatment, improved health outcomes, increased health awareness, better management of multiple health conditions, and empowerment through health knowledge as facilitators. Participants articulated a multifaceted approach to screening integration, conceptualizing leadership as a collaborative effort involving multiple stakeholders, including healthcare providers, government agencies, non-governmental organizations, HIV program coordinators, and researchers. Our finding suggests that women living with HIV (WLWH) were comfortable with trained nurses administering thermal ablation for cervical cancer, even if they had no prior knowledge of the procedure. Acceptability of cervical cancer screening integration into routine care could be high when barriers were addressed.

## Introduction

Cervical cancer poses a substantial global health challenge, disproportionately affecting low- and middle-income nations (1). Globally, cervical cancer ranks fourth in cancer incidence among adult females, and second in women aged 15–44 years (2). In West Africa, the mortality rate of cervical cancer is three times the global cervical cancer mortality rates (3). In Ghana, cervical cancer ranks second among female cancers. Data from 2023 indicate 2,797 incident cases and 1,699 fatalities. The world health organization (WHO) projects an increase to 5,000 new cases and 3,361 deaths annually by 2025, highlighting a critical public health concern (3, 4).

Screening and HPV vaccination have demonstrated efficacy in disease prevention (1). However, the high burden of cervical cancer in low- and middle-income countries is a reflection of the lack of widespread efficient screening for cervical cancer (5). Nonetheless, the integration of cervical cancer screening into routine HIV care in Ghana is imperative due to the disproportionate impact of cervical cancer on women living with HIV (6). In 2018, 5.8% of new cervical cancer cases were in WLWH, with 4.9% attributable to HIV infection (6, 7).

HIV infection increases cervical cancer risk six-folds due to immunosuppression and immune activation (7, 8). In Ghana, where over 219,986 women were living with HIV in 2019 (9), the lack of routine cervical cancer screening at HIV clinics is concerning. While antiretroviral therapy is provided, essential services like cervical cancer screening, common in developed countries, are not routinely offered in Ghanaian HIV clinics (10). Cervical cancer’s age-standardized incidence and death rate in 2022 were 27.0 and 16.9 per 100,000, respectively (11). Furthermore, some studies highlighted the incidence of HPV infection among Ghana’s various populations, a study in the North Tongu District of Ghana, found that 32.3% of the samples examined had high-risk HPV (12). Another study established that 72% of pregnant women in Ghana between 18 and 41 years had high-risk HPV genotypes with an overall HPV prevalence of 65% (13). Given that a high proportion of WLWH in Ghana are at risk for cervical cancer, qualitative interviews with people living with HIV could provide crucial insights into the barriers and facilitators for implementing cervical cancer screening, potentially informing strategies to integrate this vital service into routine HIV care.

Assessing the preferences of women living with HIV regarding self-sampling for HPV DNA testing is crucial in the Ghanaian context. While cervico-vaginal home-based self-sampling has proven effective for HPV DNA testing in various settings (14), its applicability to women living with HIV in Ghana requires careful consideration. This is because stigma remains a big issue for people living with HIV in Ghana (15). Most people live in compound houses and may not have privacy at home to self-sample. Given these constraints, women may prefer clinic-based self-sampling or provider-administered sampling. Understanding these preferences is essential for the effective integration of cervical cancer screening into routine HIV care, ensuring cultural appropriateness, and maximizing participation rate.

Healthcare researchers and policymakers now recognize that measuring endpoints of health outcomes is not enough. Rather, a critical part of implementation is to perform formative evaluations to determine how stakeholders perceive the intended intervention in a particular social and cultural context. Therefore, it is crucial to ascertain how patients feel about integrating CC screening and care into routine HIV care. We chose qualitative interviews for this because going in-depth with stakeholders to discuss reasons for their choices and recording their feelings and behaviors is likely to yield much deeper insights than simplified questionnaires.

## Methods

### Study population

The study was carried out in three administrative regions of southern Ghana, which have high prevalence of HIV, the Greater Accra, Central, and Eastern Regions. We have carefully chosen six health facilities from these regions to have a mix of urban, suburban, and rural facilities to enable us to obtain a wide spectrum of experience and implementation challenges and facilitators.

#### Korle-Bu Teaching Hospital (KBTH)

The KBTH is situated in Accra in the Greater Accra Region. It is the largest hospital in Ghana and serves as the teaching hospital for the University of Ghana Medical School. The Hospital has over 2,000 beds and 17 clinical and diagnostic Departments/Units. It has an average daily attendance of 1,500 patients and 250 patient admissions. The HIV clinic in the Department of Medicine sees over 16,000 HIV patients annually. Monthly, the clinic sees about 1,400 unique HIV patients out of which two-thirds are females and over 70% have their virus suppressed on ART (viral load undetectable). KBTH was chosen for this study because the catchment area for the clinic is cosmopolitan with people from all over the metro area of about 6 million people. In addition, this site sets the standard of care for the rest of the country.

#### Cape Coast Metropolitan Hospital

Cape Coast Metropolitan Hospital, located in the capital of the Central Region, is a major referral center providing varied clinical services, including comprehensive HIV care. The clinic recorded a total of 45 male clients spanning various age groups. The clinic serves 153 female patients, most of them aged 40 years and above. The clinic operates DSD services daily, ensuring clients can access care with flexibility. The wide age distribution and high number of older adult clients make this facility an important site for the study. Its urban setting and accessibility further enhance its value for research.

#### Elmina Polyclinic

Elmina Polyclinic, located in the Central Region of Ghana, is a key healthcare facility within the Elmina township and surrounding communities. The facility is actively involved in the provision of HIV care and treatment services. The clinic manages 478 women living with HIV under the age of 14 and an additional 6 female clients aged exactly 14 years. This highlights a significant burden of pediatric and adolescent HIV care within the facility. Elmina Polyclinic provides Differentiated Service Delivery (DSD) daily to accommodate the needs of its clients. Wednesdays are designated for static clinic days for structured patient reviews and adherence support. The clinic was chosen for this study due to its unique client demographic.

#### Ewim Polyclinic

Ewim Polyclinic is in the Central Region and involved in HIV treatment and care. At the end of February 2025, the facility had 344 clients actively receiving antiretroviral therapy (ART), comprising of 116 males and 228 females. Among the female clients, 219 are aged 20 years and above. The clinic records an average weekly attendance of 40 clients. Ewim Polyclinic offers static HIV clinic services primarily on Tuesdays and Thursdays, while Differentiated Service Delivery (DSD) is provided every day to enhance accessibility and adherence. This facility was selected for the study due to its active HIV client base and its efficient scheduling model, which facilitates regular engagement with clients.

#### St. Martin de Porres Hospital, Agomanya

The HIV treatment center at this hospital is one of the oldest in the country. There is a high burden of diseases in this district, and it was chosen for it being rural. The clinic sees about 5,000 patients per year and about 200 unique patients weekly. The NMIMR has had a long-standing relationship with this clinic. Physician Assistant Joseph Tetteh, the medical director of the clinic, oversaw the day-to-day recruitment at this site.

#### Atua Government Hospital

Atua Government Hospital, located in Manya Krobo district of the Eastern Region of Ghana, is a district hospital. The hospital is currently managing over 3,000 HIV patients. On average, the hospital sees approximately 23 HIV patients per day for clinical care and monitoring. The HIV clinic is integrated into the hospital’s broader service delivery and is staffed by a dedicated team of clinicians and nurses who provide antiretroviral therapy (ART). Atua Government Hospital was selected for this study due to its important role in HIV service delivery within a semi-urban setting and its consistent patient engagement, which makes it a valuable site for implementing and evaluating interventions.

### Population and sample size estimation

Using the CFIR to inform questions, we conducted semi-structured in-depth qualitative interviews with 85 PLWH, across all six sites to identify factors that could promote or impede the integration of cervical cancer screening into HIV care in Ghana. The tool was translated first into the three dominantly spoken local languages in southern Ghana and then back translated into English during Interviewers’ training, at least by two persons, and there were very little discrepancies, which were easily resolved with the support of a third person. The tool was also pretested, and the findings were used to revise it before the commencement of the main study. Table 1 shows how CFIR was used to guide the design of data collection tools for PLWH.

**Table 1:** CFIR domains and possible qualitative focus areas for PLWH.

CFIR domain	Information gathered
Intervention characteristics	Preference for HPV self-sampling versus provider sampling, same day test and treat versus coming to clinic another day, preference for nurse provider versus doctor provider, etc.
Inner setting	Satisfaction with current HIV care, whether adding HPV and CC care will help or hurt HIV care.
Outer setting	Assessment of trust in the healthcare system, relative confidence in abilities of healthcare professionals to identify and treat HPV/CC, vies of the healthcare staff, opinions of spouses about CC.
Individual characteristics	Knowledge about cancer and about CC. Rumours and experiences in the community, experiences with people peddling CC cure in the community.
Implementation process	Accessibility to clinic, waiting times, willingness to add HPV/CC care, perceptions of costs.

### Sample size

We aimed to have a large enough sample size to discover different opinions but not to go beyond one additional interview when saturation point is reached, and no new information was generated. We chose to interview more women living with HIV because they are the target group for the intervention. The final number of interviews conducted varied from facility to facility based on the number of clients on their register (Table 2).

**Table 2:** Number of PWH recruited.

Name of Health facility	WLWH	MLWH	TOTAL
Korle-Bu Teaching Hospital, Korle-Bu, GA/R	21	10	31
Ewim Polyclinic, Ewim, C/R	5	3	8
Cape Coast Metro Hospital, Cape Coast, C/R	5	3	8
Elmina Polyclinic, Elmina, C/R	5	3	8
Atua Government Hospital, Atua, E/R	10	5	15
St. Martin de Porres Hospital, Agomanya	11	4	15
TOTAL	57	28	85

### Selection of participants

We used purposive sampling to select participants for the study. For women and men living with HIV, we approached patients as they came to the clinic and enrolled those who agreed to participate in the study as discussed under ethics. However, to ensure the inclusion of minority opinions, we spoke with counsellors on the ground to identify specific minority patients such as men who have sex with men, or women who have sex with women, or any transgender patients, they know about so that we can embed their opinions. We have set the female-to-male ratio to 2:1 depicts Ghana’s HIV population demographics (Table 2). Although the intervention is for women only, we included men in these interviews because most men with HIV have women with HIV as partners and may therefore play a role in getting their partners to the clinic for cervical cancer care. Additionally, men were included in the study for gender balance in the views on acceptance of cervical cancer screening and its integration into HIV care as well as the acceptance of self-sampling among women. It is vital to appreciate the gender perspectives because in most traditional settings in Ghana, males, especially husbands and fathers are the key decision makers about household health seeking behaviors, where women need permission from their husbands or fathers before seeking certain kinds of health care. In all, 85 respondents (57 women and 28 men) were included in this study (Table 2).

#### Inclusion criteria

PWH were adults aged 18 to 65 years, since 64 is the age limit for CC screening and were willing to sit for 45–60 min for the interview.

#### Exclusion criteria

We excluded individuals <18 years or unwilling to consent.

### Data collection

After consenting participants, trained qualitative research assistants conducted interviews in a private area using a detailed interview guide (duration of 45–60 min). Interviews were conducted in participants’ preferred language (i.e., English, Fante, Krobo, Ewe, Twi, Ga). The interviews were audio-recorded and transcribed directly into English. Quality was ensured by having two people performed the direct transcription independently. The two transcribers then compared notes to agree on any differences between them under the supervision of the last author. There were few discrepancies between the two trained qualitative research staff. Notes were taken during data collection to complement the audio-recording. A systematic debrief with the research assistants, review of their field notes, and reading of transcripts were done by the research team as they became available. The CFIR informed the design of the semi-structured interview guides intended to explore barriers and facilitators to integrating cervical cancer screening into HIV care in Ghana and the acceptability of self-sample or provider-sample among respondents.

Qualitative analysis software, MAXQDA version 12 was used to organize the transcripts and performed analysis. Coding was done using deductive and inductive approaches in line with the CFIR adapted for this study. The inductive analysis was done by performing content analysis on the dataset to observe specific concepts and patterns in line with the thematic areas of interest. The deductive analysis starts with predetermined themes and relevance concepts that were identified while performing content analysis on the dataset. We largely performed free format coding interactively within the dataset during analysis using MAXQDA software and therefore prepared no code book beyond using the question numbers and key words from the questions as the initial thematic codes. The codes that were generated during analysis were refined through team-wide review to group codes into categories and develop themes. A narrative analysis involving reading each interview without regard to individual codes was also done to look for patterns and emerging themes within each interview. Content analysis was done on emerging themes, taking into consideration the divergent views, we selected the most representative quotes that represent majority or minority positions for presentation.

## Results

### Accessibility and availability of cervical cancer screening services

Accessibility of cervical cancer screening services was generally perceived to be difficult stemming from varied reasons. Participants cited lack of awareness, financial constraints, and the uncomfortable nature of the examination as barriers. Participants also reported that cervical cancer screening services were not readily available at health facilities closest to respondents. These views are represented in the following quotations.

*It won’t be something difficult, because when you are sick you will probably go to the hospital, and when you are screened for cervical cancer, you must go to the doctor to be treated so that you will be free from the disease.* [43-year-old woman, PLWH, EL003, IDI].

*Sometimes people do not have the money for it. Other times too it’s because of where we stay as the clinics around, we think may not help us. I believe that if they get (bring the service) into our community clinics, it will help. If someone is close to us and starts to see unusual symptoms, the person can advise us to go to the clinic for the test*. [28-year-old woman, PLWH, EW004, IDI].

### Willingness to participate in screening

Participants expressed a strong willingness to participate in cervical cancer screening. Their motivations included a desire to know their status, protect themselves, access treatment if necessary, and benefit from early detection. Some viewed screening as an opportunity for health education and prevention. Many emphasized the importance of regular check-ups and the potential for early intervention. Overall, there was a positive attitude towards screening, with participants recognizing its value in maintaining their health and well-being. A few however were skeptical on participation in the screen. These were aptly captured in the following quotes.

*Yes, I will. I want to know what is going on in my body. I must take part to know whether I’m negative or positive. Then if there is any treatment or medication, I will take it*. [42-year-old woman, PLWH, AT005, IDI].

*I’m not sure if I will partake because everything has its consequences. Where the sample is taken from doesn’t make me comfortable to participate*. [60-year-old woman, PLWH, KB002, IDI].

### Relationship with health workers

Most participants reported positive relationships with health workers. They described the staff as friendly, cheerful, and good at relating to patients. Some participants mentioned feeling comfortable bringing up their problems to the healthcare providers. Overall, the responses indicated a generally positive atmosphere in healthcare settings, which could facilitate the integration of cervical cancer screening into routine care. These were aptly captured in the following quotes.

*They are friendly with me. They are not harsh to me. I was in Kumasi before moving to Kede but Kede is far from Atua, but because the nurses here relate with us cordially, I don’t mind paying transportation cost to come here*. [40-year-old man, PLWH, AT019, IDI].

*They provide us with good services. They are always available to help us address challenges that usually affect our treatment. So, I will say I have a very good relationship with them.* [38-year-old woman, PLWH, CM006, IDI].

### Feasibility of integrating screening into routine care

There was broad consensus among participants that integrating cervical cancer screening into routine care was feasible. Many mentioned that they had been informed about it before, suggesting some level of existing awareness. Participants drew parallels to the successful integration of other services, such as HIV care, as evidence that cervical cancer screening could be similarly incorporated. These positions are captured in the quotations below.

*Yes, they can do it. They had already told us about this cervical cancer several times when we came for our medication*. [38-year-old woman, PLWH, CM006, IDI].

*From my end, I will say they will have time to do it. They relate with us in a friendly manner, counsel, and encourage us. I think they can do it. But it will depend on our women to open to them for the services. This may be a problem. But, once the women are comfortable, they can do it, because it’s their work*. [35-year-old man, PLWH, AT018, IDI].

### Impact of integration on the quality of service

Participants believed that integrating cervical cancer screening would positively impact the quality of healthcare services. They cited benefits such as early detection and treatment, improved health outcomes, and enhanced overall care. Some noted that it would complement existing services like antiretroviral treatment. Participants also mentioned that the addition of screening would demonstrate the healthcare facility’s commitment to comprehensive care. Overall, there was a strong perception that integrating cervical cancer screening would elevate the quality of healthcare services provided. The quotations below captured the views of respondents.

*It won’t affect the quality of services they provide negatively. Because these are people who have been trained and well-educated. When you give this kind of work to someone like me who is a farmer I can’t do it. So, all they must do is eat well and come and sit down*. [40-year-old man, PLWH, AT019, IDI].

*When they add screening for cancer to our clinic, it will not affect us negatively because, so long as we are alive, we shall continue to come for our medicine and checkups, and this one too is a problem that can affect our health. So, I think that it will benefit us [……]. I am told that when we have the problem they will help us to treat it and I think it would rather affect us positively, just like HIV [……], we say that it is good to know whether you have it or not, so this one too, it would be good to know whether you have it or not. [……] if you test for it and you have it, they will help you with the treatment and if you do not have it, I think they will also help you with some advice to stay free from it. So, I would not like to miss my clinic days because cancer is a serious problem so I would like to be tested for it regularly, we were taught that prevention is better than cure”* [42-year-old woman, PLWH, AT002, IDI].

### Advocacy for integrating screening into routine care

Most participants expressed willingness to advocate for integrating cervical cancer screening into routine care. They saw the value in encouraging others to participate in screening and spreading awareness about its benefits. Some participants mentioned that they would be motivated to advocate based on their own positive experiences with screening. Few, however, noted stigma as a challenge to being an advocate. These positions have been succinctly captured in the following quotations.

*Yeah, if only more education would be given to me so that if I stand there, I know what I will be talking about. I would like to go to schools, and churches to talk about cervical cancer*. [26-year-old woman, PLWH, CM001, IDI].

*I will not do it. If others will know about it, which is why I asked if this interview would be made available to the public. I don’t want the public to know about my status because HIV is a shameful disease*. [60-year-old woman, PLWH, AT002, IDI].

### Support needed to facilitate advocacy for cervical cancer screening

Participants identified several forms of support they would need to become effective advocates for cervical cancer screening. These included training on the disease, its causes, and treatment options. Some mentioned the need for resources to support community outreach, such as transportation for door-to-door advocacy. Others emphasized the importance of motivation and ongoing support.

*The training may take place at the clinic but to move from one place to the other to talk to other people may require money and I think transportation support will help*. [30-year-old man, PLWH, AG008, IDI].

*Now I don’t know much about it. So, you will have to educate me on how to go about it and support me with money if I must go to places to educate other women about cervical cancer*. [45-year-old woman, PLWH, AT017, IDI].

### Barriers to integration

Participants identified various potential barriers to integrating cervical cancer screening into routine care. These included cultural beliefs that certain diseases are supernaturally caused and therefore require supernatural treatment, lack of training for healthcare providers, stigma (both self-stigma and public stigma), lack of awareness about the importance of screening, and anxiety about test results. Some participants mentioned resource constraints, such as the need for specialized equipment or personnel. The following narratives summarize these views.

*Superstition […]. Probably the person doesn’t believe that cervical cancer exists, even though the person may have heard a lot about it. So, the person has her own opinions and doesn’t want other opinions to interfere with hers. The person might also be shy. Also, the hospitals may not have the appropriate instruments or specialists for such a job*. [26-year-old woman, PLWH, CM005, IDI].

*I will say due to lack of money, or lack of support from the government. The equipment needed for the screening and treatment may not be available, which I think is also because of a lack of funds*. [67-year-old woman, PLWH, KB032, IDI].

### Suggestions to overcome barriers

Participants offered several suggestions to overcome barriers to cervical cancer screening integration. These included comprehensive training for healthcare providers, community education to reduce stigma and increase awareness, early introduction of the screening program, and ensuring the availability of necessary resources. Some suggested integrating screening more closely with existing services, such as HIV care. The quotations below captured the views of respondents. The following narratives summarize these views.

*More education should go to the nurses. The way they have been keeping our status, they should keep this one too because stigma can kill. The cost too if you people will take half and we will also take half. Per the education, they will be willing to do it ooh but the money issue, so if some organization or you people will take half or you people will take the cost of screening and we will buy the drugs, or you people will buy the drugs, and we will pay for the screening*. [26-year-old woman, PLWH, CM001, IDI].

*They need to make announcements about cervical cancer on radio stations. Educate our women about it when they visit the clinic for their medications and routine checkups. When we do this, those women with the disease who are hiding it will start coming out. This will encourage other women*. [40-year-old man, PLWH, AT019, IDI].

### Leadership in the integration process

Participants identified various potential leaders who could facilitate the integration of cervical cancer screening process into HIV routine care. These included healthcare providers (nurses and doctors), government agencies like the Ghana AIDS Commission, NGOs, and existing HIV program coordinators. Some participants suggested that the researchers or facilitators introducing the screening program should take a leadership role. There was general recognition that leadership at multiple levels would be necessary for successful integration. These positions have been succinctly captured in the following quotations.

*The only people who understand the importance of our situation and how serious cervical cancer is and should lead the integration process is the Ghana AIDS Commission*. [67-year-old woman, PLWH, KB032, IDI].

*The nurses should be able to educate us about cervical cancer when we come for our medications. Also, assemblymen and MPs can lead this process. But in all, if the government leads this process all the challenges can be overcome*. [32-year-old woman, PLWH, CM003, IDI].

### Trust in health workers

Most participants expressed trust in their health workers. When asked about their comfort level with health workers explaining procedures, almost all participants responded positively. This trust appears to be built on past positive experiences, the quality of care received, and the health workers’ approachability. These views are captured in the quotations below.

*Yes, I trust them because of how patient they were with me when I first came here. How they took their time to take me through counselling, and these made me be at peace with myself. I did not believe it when I was first told I had HIV, but when I came here their attitude made everything simple for me*. [44-year-old woman, PLWH, KB015, IDI].

*I trust them because, at the beginning, there were lots of issues, but I discussed it with them. They insisted that we do an HIV test even though they didn’t know I was HIV positive, so I think they are the best*. [24-year-old man, PLWH, EW005, IDI].

### Satisfaction and continuous coming to the clinic

All participants expressed happiness and satisfaction with services received at the clinic and reported that they will continue to attend clinic at the various health facilities. These views are represented in the following quotations.

*Yes, I am happy to come to this facility for medication. I will come*. [54-year-old woman, PLWH, KB014, IDI].

*I am generally happy coming to this clinic, and so is my wife*. [61-year-old man, PLWH, CM002, IDI].

*I will keep coming because of the counselling services provided. How the nurses take time to explain the procedures, and everything to you, keeps you coming*. [26-year-old woman, PLWH, CM001, IDI].

### Individual preferences and acceptance

We explored participants’ preferences regarding sample collection methods, perceived challenges associated with self-sampling, and other different screening procedures as well as their views on receiving test results. We also looked at participants’ satisfaction with and acceptance of cervical cancer screening services, how they feel about healthcare providers performing procedures, and their awareness of treatment options. They have expressed satisfaction with incorporating cervical cancer screening into their regular HIV clinic visits. These views were expressed in the following quotations.

*I would prefer them to teach me, so I take the sample myself. I prefer that because I can do it in the comfort of my home since I would have been taught*. [48-year-old woman, PLWH, KB005, IDI].

*If we are trained in how to do it, I think that most people would be more comfortable doing it at home than coming to the facility for someone else to do it since it’s a private area*. [24-year-old woman, PLWH, EW011, IDI].

### Comfortable with trained nurses performing cervical cancer screening

All participants reported feeling comfortable with trained nurses performing cervical cancer screening procedures. Many cited the nurses’ training and expertise as reasons for their comfort. These views are captured in the quotations below.

*Yes, I would be comfortable for nurses at the ART clinic to be trained to do this procedure because I know them, and I trust them to other nurses from different clinics in the facility*. [38-year-old woman, PLWH, CM006, IDI].

*Yes, I prefer the trained nurses doing the procedure, because they have been trained so they know what to do*. [44-year-old woman, PLWH, EL007, IDI].

### Comfortable with trained nurses administering thermal ablation

Despite the lack of prior knowledge of thermal ablation, all participants have mentioned that they are comfortable with it when trained nurses administer it (thermal ablation) to them. Many of them cited trust in the nurses’ training and the potential health benefits of the treatment as reasons for their acceptance. The views of respondents were presented in the following narrative.

*Yes, I will. Oh, it’s just like I said, they’re helping me with my health and if this will help me to also reduce the risk, why won’t I? With me, anything concerning my health I’m ever ready to do anything*. [32-year-old woman, PLWH, EL001, IDI].

*Yes. Because they have been trained and they know what they are doing*. [26-year-old woman, PLWH, CM005, IDI].

### Acceptance by peers

Participants generally believed that their peers would accept cervical cancer screening and related procedures. Few, however, believed that some people may not accept due to individual differences. The quotations below captured the views of respondents.

*If you educate them well to understand what you are doing, I’m sure most people will accept it*. [60-year-old woman, PLWH, AT002, IDI].

*Yes, because I know the benefits of participating in screening, I think they will also accept it*. [36-year-old woman, PLWH, AG001, IDI].

### Integration of cervical cancer screening into routine services at the facility

Participants offered various suggestions for integrating cervical cancer screening into routine services. These included adding screening to regular HIV care appointments, providing comprehensive education and health talks, training ART coordinators and nurses, and ensuring the availability of necessary equipment. Some emphasized the importance of scheduling screening alongside other routine services to normalize the procedure and increase participation. The quotations below captured the views of respondents.

*It should be just like coming for the viral load test. With this, if you have the time for the viral load then you should have the time for that screening as well because it’s all helping our system just to make us live long*. [32-year-old man, PLWH, EL001, IDI].

*It would be very simple to integrate. When you come to the hospital, you go through your normal checkup routine, take your medication, and then you’ll be screened. If the disease is detected, you start treatment. So, it will be very simple because no one knows what we do here at the health facility*. [51-year-old woman, PLWH, KB042, IDI].

## Discussion

Our study provides insights into the barriers and facilitators for integrating cervical cancer screening into routine care for women living with HV through the application of the Consolidated Framework for Implementation Research (CFIR). By leveraging CFIR’s comprehensive domains, we developed targeted interview questions that explored the complex interplay of factors affecting implementation in southern Ghana. Our findings have provided insight into the dynamics of integrating cervical cancer screening into routine HIV care to improve healthcare engagement and outcomes for people with HIV.

### Process of screening

We investigated the integration of cervical cancer screening into routine HIV care, focusing on participants’ perspectives regarding screening willingness, healthcare provider relationships, and the potential feasibility of service integration. Most participants demonstrated a strong willingness to participate in cervical cancer screening. Their motivation stemmed from multiple factors, including a desire to understand their health status, protect themselves, access timely treatment, and benefit from early detection. Receiving a brief health education may increase people’s positive attitude toward cervical cancer screening (16). Screening was viewed not merely as a medical procedure but as an opportunity for health education and preventive care. Participants consistently emphasized the significance of regular check-ups and early intervention. However, the study also captured nuanced perspectives, with a few participants expressing reservations. Our study supports other findings that some people may not uptake cervical cancer screening if they are performed by male practitioners (17–19). Many participants felt sufficiently comfortable to discuss personal health concerns openly with their healthcare providers. This suggests that healthcare workers’ actions and attitudes can shape patient perceptions of healthcare services (20).

Participants believed that integrating cervical cancer screening into routine care is feasible. Many were already aware of the concept, indicating existing groundwork for implementation. Participants drew parallels with successful service integrations, such as existing HIV care models. This is in line with another study that claimed that the integration of the HPV vaccine with other services is an established priority, but programmatic evidence is needed in LMICs (21). Participants believed that integrating cervical cancer screening would positively impact healthcare service quality. They identified multiple potential benefits, including enhanced early detection and treatment capabilities, improved health outcomes, and more comprehensive care. The proposed integration was seen as a complementary approach to existing services like HIV treatment. Participants viewed the potential screening integration as a demonstration of the healthcare facility’s commitment to holistic patient care. These findings suggest a promising foundation for integrating cervical cancer screening into routine HIV care, underpinned by patient willingness, positive healthcare relationships, and perceived potential for improved health outcomes.

### Implementation outcomes

The study explored the perspectives of people living with HIV (PLWH) regarding the integration of cervical cancer screening into routine HIV care, revealing complex insights into potential implementation strategies, perceived benefits, and challenges. Participants demonstrated a comprehensive understanding of the potential health benefits associated with cervical cancer screening, articulating multiple advantages, including early detection and treatment, improved overall health management, and the potential for extended life expectancy. This is in line with a review paper that has proven that testing leads to early detection and has reduced cancer rates by 50–80% (22). Beyond immediate health outcomes, they recognized the broader implications of screening, such as encouraging healthier behaviors, providing psychological reassurance, and ultimately leading to more positive health trajectories. The participants exhibited a notable commitment to advocacy for cervical cancer screening integration, with most expressing a strong willingness to promote screening within their communities, while simultaneously recognizing persistent stigma and knowledge gaps as substantial barriers to effective community engagement. Our study supports another study in China where it was found that knowledge is a predictor for willingness to participate in screening (23). Participants also emphasized the critical need for comprehensive training, resource allocation, and ongoing support to enable meaningful advocacy.

The research identified a nuanced landscape of facilitators and barriers to cervical cancer screening integration. Positive facilitators included the potential for early detection and treatment, improved health outcomes, increased health awareness, better management of multiple health conditions, and empowerment through health knowledge. Conversely, reported in other studies, significant barriers encompassed cultural beliefs and misconceptions, limited healthcare provider training, persistent social stigma, low awareness about importance of screening (23, 24), patient anxiety about test results (24–26), and resource constraints (23), still exist and must be addressed to enhance intervention uptake. Unlike other studies, we did not find inequalities in access to screening in terms of physical disabilities, lack of digitalization, residing in highly deprived communities (27), and survivors of sexual violence (28). This could be because we did not specifically probe for them, therefore, program implementers must be aware of these potential barriers and be ready for them.

Participants articulated a multifaceted approach to screening integration, conceptualizing leadership as a collaborative effort involving multiple stakeholders, including healthcare providers, government agencies, non-governmental organizations, existing HIV program coordinators, and research facilitators and this supports what was reported in another study that when medical heads and coordinators are jointly in charge of an intervention, it improves adherence (29). Participants emphasized that the implementation of integrating cervical cancer screening into existing HIV care services may be seamless through comprehensive healthcare provider training and this confirmed what was reported in another study (29).

### Individual characteristics

The study also explored the acceptance and preferences of participants regarding cervical cancer screening procedures during their HIV clinic visits. Participants expressed a strong willingness to undergo cervical cancer screening during their regular visits for antiretroviral therapy (ARVs) which is in line with other studies (30, 31). They highlighted the convenience of incorporating the screening into their existing healthcare routine. Participants also demonstrated comfort with trained nurses performing the procedures, emphasizing the nurses’ expertise and the established trust within the clinic setting. Despite being comfortable with Provider sampling, participants also reported that they will accept self-sampling when they are trained in how to do it because they will be more comfortable taking the sample by themselves privately. However, a significant finding revealed a lack of awareness among participants regarding thermal ablation as a treatment option for precancerous cervical lesions. Many participants reported being unfamiliar with this treatment modality, suggesting a potential need for increased education and awareness-raising efforts.

### Service outcome

Our finding suggests that people with HIV (PWH) were comfortable with trained nurses administering thermal ablation for cervical cancer, even if they had no prior knowledge of the procedure. They trusted the nurses’ training and recognized the potential health benefits. The PWHs generally believed that their peers would accept cervical cancer screening and related procedures. This corroborates with a study in Malawi which found motivation from peers as driver for cervical cancer screening (31). Our findings show that with proper education about the benefits, most people would accept the procedures, although individual differences might lead to some people rejecting the procedures.

## Conclusion

This qualitative study shows that the CFIR is a useful tool that can be used to inform the design and implementation of interventions that consider facilitators and barriers, which could be addressed to enhance the achievement of intervention outcomes. Our study reveals that women will screen for cervical cancer because of the desire to know their health status, protect themselves, access timely treatment, and benefit from early detection. The study also suggests that more women with HIV (WWH) may accept the screening performed by female health personnel. We also found that multiple stakeholders’ engagements with the health sector, academia, and NGOs will facilitate a smooth integration of cervical cancer screening into routine care for WWH. Thus, acceptance of integrating cervical cancer screening into routine care is very high, however, this requires that healthcare providers will treat clients with respect, provide timely information to the clients and assure them of confidentiality Also, self-sampling must be tested and when found to provide comparable results with provider-sampling, then clients must be trained to do it, this will take away the time for sampling at the health facility by care providers and thereby shorten the time spent at the clinic.

## Data Availability

The raw data supporting the conclusions of this article will be made available by application to the corresponding authors after removing all personal identifiers, without undue reservation.
